# Comparative Evaluation of Pesticidal Potential of Five Aromatic Plants, with Emphasis on the Fungicidal Activity of *Lavandula dentata* and *Thymus vulgaris* Extracts Against the Soil-Borne Tomato Pathogens *Fusarium oxysporum* f.sp. *radicis-lycopersici* and *Verticillium dahliae*

**DOI:** 10.3390/microorganisms14051001

**Published:** 2026-04-29

**Authors:** Aikaterini Gropali, Ioannis Stavrakakis, Nikolaos Remmas, Shereen Basiouni, George Tsiamis, Asma Ben Salem, Salma Lasram, Mete Yilmaz, Mevlut Emekci, Fatma Acheuk, Awad A. Shehata, Wolfgang Eisenreich, Paraschos Melidis, Spyridon Ntougias

**Affiliations:** 1 Department of Environmental Engineering, Democritus University of Thrace, Vas. Sofias 12, 67132 Xanthi, Greece; agropali@env.duth.gr (A.G.); istavrak@env.duth.gr (I.S.); nremmas@env.duth.gr (N.R.); pmelidis@env.duth.gr (P.M.); 2 Cilia Cell Biology, Institute of Molecular Physiology, Johannes Gutenberg University, 55128 Mainz, Germany; shereenbh@yahoo.com; 3 Laboratory of Systems Microbiology and Applied Genomics, Department of Sustainable Agriculture, University of Patras, 2 Seferi St, 30100 Agrinio, Greece; gtsiamis@upatras.gr; 4 Laboratory of Molecular Physiology of Plants, Center of Biotechnology of Borj Cedria (CBBC), BP 901, Hammam-Lif 2050, Tunisia; asma.bensalem@cbbc.rnrt.tn (A.B.S.); salma.lasram.cbbc@gmail.com (S.L.); 5 Department of Bioengineering, Bursa Technical University, Bursa 16310, Türkiye; mete.yilmaz@btu.edu.tr; 6 Department of Plant Protection, Faculty of Agriculture, Ankara University, Keçioren, Ankara 06135, Türkiye; mevlut.emekci@agri.ankara.edu.tr; 7 Laboratory for Valorization and Conservation of Biological Resources, Faculty of Sciences, University M’Hamed Bougara of Boumerdes, Boumerdes 35000, Algeria; fatma.acheuk@yahoo.fr; 8 Structural Membrane Biochemistry, Bavarian NMR Center, Technical University of Munich (TUM), 85748 Garching, Germany; awad.shehata@tum.de (A.A.S.); wolfgang.eisenreich@mytum.de (W.E.)

**Keywords:** *Lavandula dentata*, *Thymus vulgaris*, botanical pesticide, fungicide agent, plant and environmental protection, biorefining value pyramid

## Abstract

The transition toward a sustainable agri-food system, aligned with agricultural and environmental policy objectives, has increased interest in aromatic plants as non-synthetic pesticide alternatives. This study focused on evaluating the antifungal potential of five specific aromatic plant species, particularly *Lavandula dentata*, *Origanum vulgare*, *Thymus vulgaris*, *Salvia officinalis* and *Rosmarinus officinalis*, against the phytopathogenic soil-borne fungi *Fusarium oxysporum* f.sp. *radicis-lycopersici* and *Verticillium dahliae*. During screening, *L. dentata* and *T. vulgaris* extracts exhibited strong in vitro fungicidal activity. Bioactive compounds previously detected in both lavender and thyme were identified in their extracts using a triple quadrupole/linear ion trap mass spectrometer. Assessment of in vitro phytoprotective action of *L. dentata* extract in solid and liquid growth media demonstrated inhibitory effects against *F. oxysporum* f.sp. *radicis-lycopersici* at concentrations above 1% *v*/*v*, with inhibitory effects of *L. dentata* extract being observed at concentrations equal to or above 2% *v*/*v*. *T. vulgaris* extract inhibited *V. dahliae* growth on solid media at concentrations at 1% *v*/*v* or above, while inhibitory effects were observed in broth media containing 2% *v*/*v* thyme extract. Seed germination tests of both *L. dentata* and *T. vulgaris* revealed a concentration-dependent reduction in their germination index (GI) at concentrations equal or above 2% *v*/*v*, apart from the effect of lavender extract on cress, where inhibition occurred at dose application above 5% *v*/*v*. In planta experiments demonstrated the complete phytoprotective action of lavender extract against *F. oxysporum* f.sp. *radicis-lycopersici*, while a marginal improvement in plant survival was observed during application of *T. vulgaris* extract.

## 1. Introduction

The increase in the global population, which is expected to rise from 8.28 billion in 2026 (https://www.worldometers.info/world-population/; accessed at 7 April 2026) to 10.3 billion people in 2080 [1], requires a corresponding rise in agricultural production to satisfy the growing nutritional needs. Thus, agricultural practices should be optimized to increase crop productivity and minimize production losses. In this direction, total pesticide use has increased by 14% compared with the last decade, reaching a total production of 3.73 million tonnes (Mt) of active substrates in 2023 [2].

The Farm to Fork Strategy (F2F) under the European Green Deal (2020) intends to reduce chemical pesticides by 50% in the year 2030, strengthening the transition to sustainable agriculture and organic farming practices. The extended use of biopesticides can be an alternative approach to achieve F2F targets, with the FAO noting “the rising importance of biopesticides as non-chemical alternatives” [3]. Biopesticides have been reported to represent 10% of the world’s pesticide market [4]. Although botanical pesticides have been applied for many years in agriculture, they have gained attention in the last decade for use in sustainable agriculture since they are considered eco-friendly, biodegradable, bioactive substrates with target specificity [5]. Botanical pesticides derived from plant-based compounds are widely available [6], representing a valuable alternative to conventional synthetic pesticides [7]; however, obstacles for their use should be considered [5]. In addition, plant secondary metabolites, including alkaloids, phenolics, terpenes and flavonoids, can exhibit antifungal activity through various mechanisms [8]. Kai et al. [9] reported that chlorogenic acid, a plant derived polyphenol with antioxidant and antimicrobial properties, significantly inhibited *Fusarium fujikuroi* in cherry tomato by suppressing conidial germination, germ tube elongation, cell viability and mycelial growth. Yörük [10] reported that the flavonoid rutin hydrate inhibited the growth of *Fusarium graminearum* that infects cereals. Furthermore, gallic acid and its derivatives syringic and pyrogallic acids efficiently reduced disease symptoms caused by *Alternaria solani* on tomato plants [11].

*Lavandula dentata* L. is a plant that naturally flourishes in the Mediterranean basin, and its essential oils and extracts have a variety of applications, such as medicinal, antifungal, antioxidant and anti-inflammatory, due to their chemical composition [12], which includes flavonoids, terpenoids and tannins [13]. Numerous studies have demonstrated the antibacterial, insecticidal, and antifungal properties of *L. dentata*. Vicenço et al. [14] reported that a 0.6% *v*/*v* concentration of *L. dentata* essential oil resulted in 100% mortality of *Anticarsia gemmatalis* after 24 h, a pest responsible for significant damage to soybean crops. Furthermore, *L. dentata* essential oil at 40 µL/L air demonstrated a mortality rate of 82.5% against *Callosobruchus maculatus* beetles [15]. In addition to its insecticidal action, Aboulwafa et al. [16] highlighted its effectiveness in inhibiting the biofilm formation of the pathogenic bacterium *Pseudomonas aeruginosa* at a concentration of 0.625 mg/mL. Wagner et al. [17] highlighted the fungicidal properties of *L. dentata* essential oils against key plant pathogenic fungi, including *Cercospora kikuchii*, *C. sojina*, and *Septoria glycines*, suggesting that *L. dentata* essential oils could be an effective addition to future integrated pest management strategies. Studies by El Abdali et al. [12] and Rahmouni et al. [18] demonstrated the inhibitory effects of *L. dentata* on *Fusarium oxysporum*. However, studies specifically addressing the interaction between *L. dentata* and *F. oxysporum* remain limited, with Si Mohammed et al. [19] reporting in vitro inhibitory activity of *L. dentata* against *F. oxysporum* f.sp. *radicis-lycopersici*.

*Thymus vulgaris*, a member of Lamiaceae family, is native to the Mediterranean region. *Thymus* species are known for their antioxidant, antibacterial, antifungal, and in-secticidal properties due to their chemical composition, containing flavonoids, phenolics, saponins and tannins [20]. Casas et al. [21] found that *T. vulgaris* essential oil at 15 μL/mL resulted in effective aphid management, reporting 78.3% mortality of *Rhopalosiphum padi*. The antibacterial properties of *T. vulgaris* against both Gram-positive and Gram-negative pathogenic bacteria, such as *Staphylococcus aureus*, *Escherichia coli*, *P. aeruginosa*, and *Klebsiella pneumoniae*, have been well-reported [20,22,23]. Jung et al. [24] tested *T. vulgaris* essential oil and its constituents, thymol and carvacrol, against *Agrobacterium tumefaciens*, reporting minimum inhibitory concentrations (MICs) of 800 μg/mL, 350 μg/mL and 350 μg/mL, respectively. Furthermore, *T. vulgaris* essential oil completely inhibited the mycelial growth of phytopathogens *F. oxysporum* f.sp. *radicis-Iycopersici*, *Phytophthora infestans*, and *Rhizoctonia solani* [25]. Aoujil et al. [26] assessed the inhibitory effect of *T. vulgaris* essential oil on *Botrytis cinerea* mycelial growth, whereas both essential oils and extracts of *Thymus* species and especially *T. vulgaris* have exerted suppressive action against *Verticillium dahliae* [27,28,29]. Giamperi et al. [29] reported in vitro complete inhibition of *Phytophthora cinnamomi*, *Pyrenochaeta lycopersici*, and *V. dahliae* during application of 400 ppm *T. vulgaris* essential oil. *T. vulgaris* essential oil, at an application dose of 0.25 mg/L, inhibited the mycelial growth of *V. dahliae* by 90% [30].

To our knowledge, there is limited information in the international literature on in vitro studies and no available data on in planta assays using *Solanum lycopersicum*, regarding the effects of *L. dentata* and *T. vulgaris* extracts on the soil-borne pathogens *Fusarium oxysporum* f.sp. *radicis-lycopersici* and *Verticillium dahliae*, respectively.

In this study, extracts of *Lavandula dentata*, *Origanum vulgare*, *Thymus vulgaris*, *Salvia officinalis* and *Rosmarinus officinalis* were comparatively evaluated for their inhibitory effects against the phytopathogenic fungi *Fusarium oxysporum* f.sp. *radicis-lycopersici* and *Verticillium dahliae*. The aim of this study was to assess the pesticidal potential of these aromatic plants through in vitro growth inhibition tests, in planta bioassays for the evaluation of their suppressiveness and phytotoxicity assessment of their extract, in order to provide effective botanical alternatives to synthetic pesticides.

## 2. Materials and Methods

To investigate the potential fungicidal activity of aromatic plants of the family Lamiaceae against the phytopathogens *F. oxysporum* f.sp. *radicis-lycopersici* and *V. dahliae*, ethanolic extracts of *Lavandula dentata*, *Origanum vulgare*, *Thymus vulgaris*, *Salvia officinalis* and *Rosmarinus officinalis* plants were prepared via Soxhlet distillation (Lenz Laborglas GmbH & Co., Wertheim am Main, Germany). These tested aromatic plants were purchased and selected on the basis of their wide availability in the Mediterranean countries. In particular, 16 g of dry matter of each tested plant (both leaves and stems) was placed in a cellulose thimble, transferred into a Soxhlet extractor containing 150 mL of absolute ethanol, heated at 80 °C under reflux until complete discoloration, dried in a rotary evaporator (Rotavapor R-200, BÜCHI Labortechnik AG, Flawil, Switzerland) and re-dissolved in 40 of mL absolute ethanol for downstream analyses. For seed phytotoxicity tests, the dried residue was resuspended in 40 mL distilled water.

### 2.1. In Vitro Screening of the Antifungal Activity of Aromatic Plant Extracts Against Fusarium oxysporum f.sp. radicis-lycopersici and Verticillium dahliae

Phytopathogenic fungi were provided by Dr. Nektarios Kavroulakis, Director of Research at the Institute of Olive Tree, Subtropical Crops and Viticulture of ELGO-DIMITRA. Mycelial agar plugs of 5 mm diameter obtained from actively growing cultures of *F. oxysporum* f.sp. *radicis-lycopersici* and *Verticillium dahliae* were placed in Petri dishes containing PDA. An amount of 50 mL of Potato Dextrose Broth (PDB) placed in conical flasks sealed with hydrophobic cotton plugs, containing 2% *v*/*v* ethanolic extract (prepared as described above) of the tested aromatic plants were used to assess in vitro potential fungicidal effects against *F. oxysporum* f.sp. *radicis-lycopersici* and *V. dahliae*. PDB alone was used as the blank, whereas PDB containing 2% *v*/*v* absolute ethanol served as the solvent control. Addition of ethanolic extract or absolute ethanol in the sterilized PDB was performed aseptically at room temperature. To assess fungicidal activity of ethanolic extracts of the tested aromatic plants, PDB alone (blank) and PDB with ethanol (solvent control) were inoculated with 100 μL of an actively growing culture of *F. oxysporum* f.sp. *radicis-lycopersici* and *V. dahliae* and incubated in shaking incubators at 120 rpm for 10 days at 25 °C. Mycelial biomass at the end of the incubation period was collected using a 0.45 μm pore-size filter and determined after drying at 70 °C. All experimental samples were examined in triplicate.

### 2.2. In Vitro Assessment of Inhibitory Effects of Lavandula dentata and Thymus vulgaris Ethanolic Extracts

**Figure 1:**
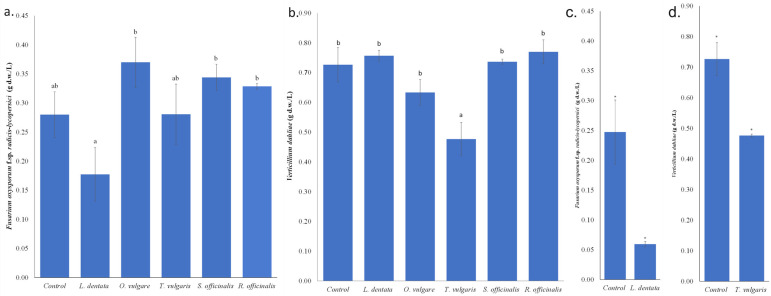
I. Effects of 2% *v*/*v* ethanolic extracts from five aromatic plants on the growth of *Fusarium oxysporum* f.sp. *radicis-lycopersici* (**a**) and *Verticillium dahliae* (**b**) in PDB medium. Bars sharing no common letter indicate statistically significant differences at *p* < 0.05 (ANOVA followed by Duncan’s multiple range test) (lowercase letters). Reassessment through an additional experiment of the effect of 2% *v*/*v* ethanolic extract of *Lavandula dentata* on *Fusarium oxysporum* f.sp. *radicis-lycopersici* (**c**), and *Thymus vulgaris* on *Verticillium dahliae* (**d**) growth in PDB medium. Student’s *t*-test was performed to assess differences in phytopathogen growth between each ethanol-containing control and the corresponding medium containing the tested ethanolic extract at the same ethanol concentration (*, *p* < 0.05). Error bars represent the standard error of mean (n = 3). Regression analysis: Growth of *Fusarium oxysporum* f.sp. *radicis-lycopersici* versus growth of *Verticillium dahliae*, R = 0.121; R^2^ = 0.015; ns, not significant.

Extracts from *L. dentata* and *T. vulgaris*, which exhibited the highest antifungal activity among the aromatic plant extracts tested (see Figure 1), were further studied to determine their inhibitory effects. PDB alone (blank), PDB with 1, 2, 3, and 4% *v*/*v* ethanol (solvent controls), and PDB containing 1, 2, 3, and 4% *v*/*v* ethanolic extracts of *L. dentata* or *T. vulgaris* were prepared. All PDB media, with or without ethanolic extract/ethanol, were inoculated with 100 µL of an actively growing culture of *F. oxysporum* f.sp. *radicis-lycopersici* and *V. dahliae*. Each fungus was tested in parallel for 10 days at 120 rpm and 25 °C. Mycelial biomass was measured as described above.

The inhibitory effects of *L. dentata* or *T. vulgaris* extracts were also evaluated on solid growth media. Potato Dextrose Agar (PDA) containing 1, 2, 3, 4, 5, 6, 7, and 8% *v*/*v* ethanolic extracts of *L. dentata* or *T. vulgaris* was prepared, together with PDA alone (blank) and PDA with 1, 2, 3, 4, 5, 6, 7, and 8% *v*/*v* ethanol (solvent controls). PDA was sterilized at 1.1 atm for 20 min, and the ethanolic extracts or ethanol were added after the medium cooled to 45 °C. Mycelial agar plugs of 5 mm diameter were used to inoculate the media, which were incubated at 25 °C. Radial growth on the different PDA media was recorded daily.

### 2.3. Germination Index (GI) Tests

The phytotoxicity of *L. dentata* or *T. vulgaris* extracts at various dilutions was recorded in seed germination tests performed in triplicate. Seed germination indices (GIs) were assessed using commercially available *Solanum lycopersicum* cv “Ace 55 VF” and *Lepidium sativum* var. *sativum* seeds. A total of 25 seeds were placed on triple filter paper and soaked with 3 mL of 1, 2, 5, 10, 12.5, 20, 25, and 50% *v*/*v* aqueous extracts of *L. dentata* or *T. vulgaris*, while blanks consisted of an equal amount of distilled water. Following incubation in the dark at 25 °C, GIs were calculated according to Zucconi et al. [31].

### 2.4. In Planta Assessment of Phytoprotective Activity of Lavandula dentata and Thymus vulgaris Extracts Against Fusarium oxysporum f.sp. radicis-lycopersici and Verticillium dahliae

The phytoprotective activity of *L. dentata* and *T. vulgaris* extracts was evaluated in planta using tomato plants. Tomato plants were grown in 400 cm^3^ pots filled with peat. Pot media were initially supplemented with CaCO_3_ to adjust the pH to 7, whereas 10 mL 0.8 g/L NPK fertilizer (20:20:20) were added weekly in each pot. Seven organic tomato seeds (*Solanum lycopersicon* cv. ACE55) were placed in each pot. An amount of 10^5^ conidia of *F. oxysporum* f.sp. *radicis-lycopersici* or *V. dahliae* per cm^3^ of peat medium was added in all pot treatment setups, excluding the ethanol and plant extract controls. No infection was observed in non-inoculated plants (healthy control), whereas complete plant severity was recorded for pathogen-inoculated plants without treatment (pathogen-only control). Pots inoculated with *F. oxysporum* f.sp. *radicis-lycopersici* and *V. dahliae* were placed in growth chambers at 18 °C and 25 °C, respectively, in a randomized design under a 16 h:8 h light:dark photoperiod. Pots used for assessing fungicidal activity were treated once per week by directly applying 0.5 mL of lavender or thyme ethanolic extract per pot early in the morning, starting from the seed stage and continuing until the end of the experiment. The cultivated plants were irrigated with water, and survival rate was assessed by recording living plants, whereas complete wilting indicated plant death. All experimental setups were based on four replicates (pots).

### 2.5. Lavandula dentata and Thymus vulgaris Extract Analysis

Prior to LC–MS/MS analysis, samples were prepared using a QuEChERS kit (QECH-D22-050, Branchia, Labbox, Barcelona, Spain), following the EN 15662 method issued by the European Committee for Standardization (https://www.cencenelec.eu/european-standardization/, accessed on 27 February 2026). Initially, 10 mL of plant extract was thoroughly mixed with 10 mL of acetonitrile containing 1% *v*/*v* acetic acid, and the QuEChERS extraction salt mixture was added. The mixture was vortexed for 2 min to enhance phase separation and subsequently centrifuged at 4000 *g* for 5 min. The organic phase was collected and diluted at a 1:1 *v*/*v* ratio with ultrapure water containing 0.1% *v*/*v* formic acid (LC–MS grade, Merck KGaA, Darmstadt, Germany). The sample was filtered through a 0.22 μm syringe filter and then directly subjected to analysis. Chromatographic analysis of lavender and thyme extracts was performed using a SCIEX ExionLC™ system coupled to a SCIEX QTRAP^®^ 4500 triple quadrupole/linear ion trap mass spectrometer (SCIEX, Concord, ON, Canada) equipped with a TurboSpray™ electrospray ionization (ESI) source and a Fortis SpeedCore C18 core-shell column (100 × 2.1 mm, 2.6 µm; Fortis Technologies, Neston, UK) operated at 35 °C under a flow rate of 0.4 mL min^−1^. The mobile phase consisted of (A) water with 0.1% *v*/*v* formic acid and 10 mM ammonium formate, and (B) methanol with 0.1% *v*/*v* formic acid and 10 mM ammonium formate. A binary gradient was used as follows (%B): 3% at 0.00 min, increased to 100% by 11.28 min, then set to 3% and held for re-equilibration until 14.10 min. The mass spectrometer was operated in scheduled multiple reaction monitoring (MRM) mode (cycle time 1.0 s) in positive ESI. Data acquisition and processing were performed using SCIEX Analyst^®^ software (version 1.7.3 HotFix 1).

### 2.6. Statistical Analysis

Analysis of variance (ANOVA) was performed using IBM SPSS Statistics v.29.0.1 software, based on Duncan’s multiple comparison test (*p* < 0.05), in order to assess significant differences among treatment setups. Regression analysis was also carried out using IBM SPSS Statistics v.29.0.1 package. Statistically significant differences between each treatment setup and the control at the same lavender or thyme concentration were identified using Student’s *t*-test at *p* < 0.05. Standard errors were calculated for all mean values.

## 3. Results and Discussion

### 3.1. In Vitro Screening of the Antifungal Activity of Aromatic Plant Extracts Against Fusarium oxysporum f.sp. radicis-lycopersici and Verticillium dahliae

Among the examined plant extracts, an inhibition of the growth of *F. oxysporum* f.sp. *radicis-lycopersici* in PDB was observed in the case of *L. dentata* ethanolic extract, which, however, was marginally not statistically significant based on Duncan’s multiple range test. This indicates phytopathogen selectivity in plant protection, probably attributed to differences in the composition of the tested plant extracts and the tolerance of the specific phytopathogens tested. Re-assessment of the inhibitory effect of lavender ethanolic extract compared to the control (PDB containing 2% *v*/*v* ethanol) showed statistically significant differences using Student’s *t*-test, for *p* < 0.05 (Figure 1a). In the case of *T. vulgaris* ethanolic extract, a significant inhibitory effect (*p* < 0.05) was exhibited against the fungus *V. dahliae* in PDB, as confirmed by both Duncan’s multiple range test and Student’s *t*-test (Figure 1b), where the growth of *V. dahliae* was inhibited by approximately 35%.

### 3.2. In Vitro Assessment of Inhibitory Effects of Lavandula dentata and Thymus vulgaris Ethanolic Extracts

**Figure 2:**
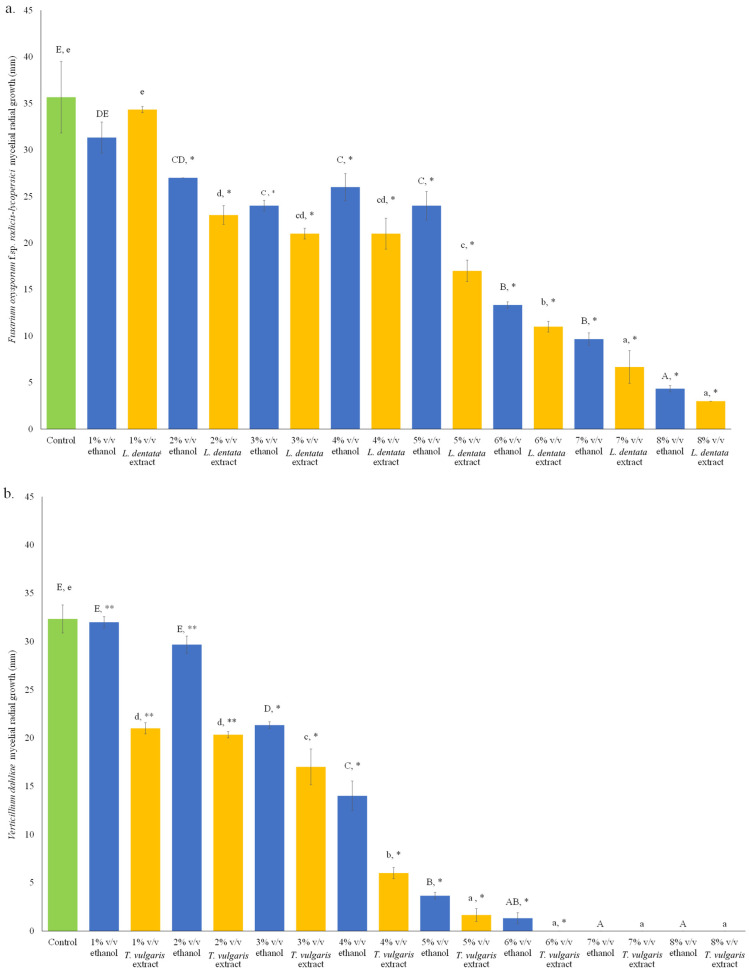
In vitro assessment of inhibitory effects of *Lavandula dentata* and *Thymus vulgaris* ethanolic extracts during the growth of *Fusarium oxysporum* f.sp. *radicis-lycopersici* (**a**) and *Verticillium dahliae* (**b**) on PDA. Control: phytopathogen growth on PDA without ethanol or plant extracts. Ethanol (1–8% *v*/*v*): growth with ethanol only. Extracts (1–8% *v*/*v*): growth with corresponding *L. dentata* or *T. vulgaris* ethanolic extracts. Bars sharing no common letter indicate significant differences (*p* < 0.05; ANOVA with Duncan’s multiple range test): lowercase for ethanol controls; uppercase for extracts. Asterisks denote differences between ethanol controls and corresponding extract treatments (*, *p* < 0.05; **, *p* < 0.01; in Student’s *t*-test). Error bars represent standard error (n = 3). Regression analysis between ethanol and corresponding ethanolic extract treatments for each phytopathogen: (i) *F. oxysporum* f.sp. *radicis-lycopersici* (ethanol vs. *L. dentata* ethanolic extract: R = 0.956; R^2^ = 0.901; *p* < 0.001); (ii) *V. dahliae* (ethanol vs. *T. vulgaris* ethanolic extract: R = 0.981; R^2^ = 0.962; *p* < 0.01).

During the application of 1% *v*/*v L. dentata* ethanolic extract, no inhibition was detected compared to the respective ethanolic and non-ethanolic controls. At concentrations of 2–4% *v*/*v L. dentata* ethanolic extract, *F. oxysporum* f.sp. *radicis-lycopersici* inhibition occurred compared to the control, although no statistically significant differences were identified among these ethanolic lavender extract treatments. On the other hand, *L. dentata* ethanolic extract setups differed significantly compared to their respective ethanol-containing cultures. A decreasing growth of this phytopathogen was observed under the application of 5–8% *L. dentata* ethanolic extract, with ethanol-containing cultures exhibiting a lower inhibitory effect than the respective lavender ethanolic extract setups. Even though limited information exists in the international literature on ethanolic extracts, ethanol is considered a “green,” biocompatible solvent that is easily biodegradable, produced through fermentation, capable of extracting a broad spectrum of bioactive compounds, and suitable for scale-up applications [32]. The above-mentioned results indicated the inhibitory effect of lavender at concentrations above 1% *v*/*v* (Figure 2a). Although various studies have been carried out on the beneficial effect of lavender against fungal phytopathogens [33,34,35,36], previous reports on the inhibitory action of this botanical fungicide on fusaria are limited. The inhibition effect of *L. dentata* hydrodistilled extract was recently examined against the wild-type *F. oxysporum* f.sp. *radicis-lycopersici* strains IB19501 and IB19502, reporting a partial inhibition at extract concentrations of 0.25 and 0.50 μL/mL PDA, whereas complete inhibition was observed at a dose application of 1 μL/mL PDA [19]. On the other hand, El Abdali et al. [12] reported that *F. oxysporum* was less sensitive to *L. dentata* hydrodistilled extract on Czapek-Dox agar. This is in accordance with the findings of Özcan et al. [37], who identified an extended lag phase at the early incubation stage of *F. oxysporum* growing on PDA in the presence of 40 ppm *Lavandula stoechas* essential oil. However, this effect was attenuated at the late incubation period. These findings are consistent with those of Marchidan et al. [38], who reported that lavender essential oil obtained by hydrodistillation from the George 90 cultivar, *Lavandula latifolia* and *Lavandula angustifolia*, completely inhibited the growth of *Fusarium culmorum* FC46 on PDA containing 40 μL essential oil/plate during the first five days of incubation. At the same time, *F. oxysporum* f.sp. *radicis lycopersici* ZUM2407 showed no significant differences in treatments applying 30 μL and 40 μL lavender essential oil/plate, while after 12 days of incubation, the growth of this phytopathogen was inhibited by approximately 80%. *L. dentata* essential oil dissolved in dimethyl sulfoxide (DMSO) exhibited an inhibitory effect against *F. oxysporum* f.sp. *albedinis*, recording MIC (minimum inhibitory concentrations of spore germination after 48 h incubation) and MFC (minimal fungicidal concentration of the mycelial growth after 3 days incubation) values of 33.3 and 40 μL/mL, respectively [18]. In addition, *F. oxysporum* f.sp. *albedinis* grown on PDA was totally inhibited by hydrodistilled *L. dentata* essential oil at a concentration of 2.5 g/L [39]. Moreover, *Lavandula angustifolia* essential oil (1.5%) inhibited the growth of *F. solani* by 97.6%, compared to the control (PDA in the absence of essential oil) [40]. Application of *L. angustifolia* essential oil on sorghum grains prior to the infection with *F. solani* led to disease severity levels similar to chemical fungicide treatment.

In control cultures containing 1% and 2% *v*/*v* of ethanol, no inhibitory effects were observed compared to the growth of *V. dahliae* on PDA media without ethanol. Ethanol concentrations of 3% *v*/*v* and higher resulted in proportional suppression of fungal growth. The application of *T. vulgaris* ethanolic extract at 1% *v*/*v* and above showed statistically significant inhibition compared to both non-ethanol and ethanol-treated cultures. The strongest inhibition was recorded at 5–8% *v*/*v*, where the thyme extracts exhibited greater antifungal activity than the respective ethanol controls (Figure 2b). Regarding antifungal properties of thyme against *Verticillium*, Giamperi et al. [29] reported complete inhibition of *V. dahliae* grown on PDA and Sabouraud Dextrose Agar in the presence of *T. vulgaris* essential oil, estimating a MIC of 325 μg/mL and a MFC of 0.60 μg/mL. Similarly, hydrodistilled *Thymus satureioides* essential oils inhibited the mycelial growth of *V. dahliae* on PDA at 16 μL essential oil per plate [28]. Rus et al. [30] reported that hydrodistilled *T. vulgaris* essential oils completely suppressed the growth of *V. dahliae* on CYGA media at a dose of 0.5 mg/L. *T. vulgaris* extract at 5 mg/mL showed a strong inhibitory effect on *V. dahliae* pathotypes V004 and V117 by 75% and 100%, respectively (grown on PDA), while *Thymus* essential oil at 5 mg/mL completely inhibited *V. dahliae* growth on PDA [27]. In accordance with these results, Erdogan et al. [41] showed the inhibitory effect of *T. vulgaris* commercial plant extract and essential oil on the mycelial growth of *V. dahliae* strains (grown on PDA) by 43–48% and 100%, at dose applications of 10 and 0.8% *v*/*v*, respectively. Water-distilled *T. vulgaris* and *T. serpyllum* essential oils showed complete inhibition of the growth of *V. dahliae* on PDA at doses of 4 and 8 μL per plate, respectively [42]. Şimşek [43] reported LC50 of 371.43 and 267.34 ppm for *V. dahliae* grown on PDA during application of *T. pectinatus* extract and *T. pectinatus*-containing AgNP (silver nanoparticles), respectively.

**Figure 3:**
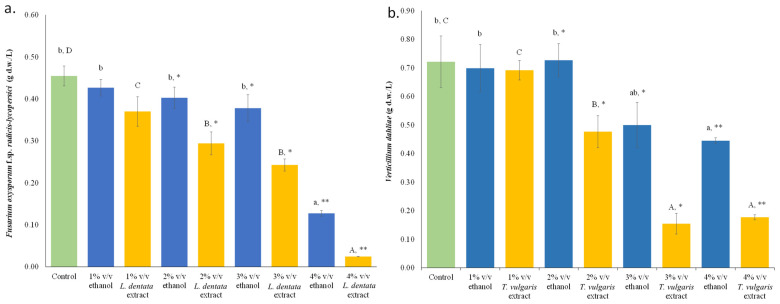
In vitro assessment of inhibitory effects of *Lavandula dentata* and *Thymus vulgaris* ethanolic extracts during growth of *Fusarium oxysporum* f.sp. *radicis-lycopersici* (**a**) and *Verticillium dahliae* (**b**) in PDB. Control: phytopathogen growth in PDB without ethanol or plant extracts. Ethanol (1–4% *v*/*v*): growth with ethanol only. Extracts (1–4% *v*/*v*): growth with corresponding *L. dentata* or *T. vulgaris* ethanolic extracts. Bars sharing no common letter indicate significant differences (*p* < 0.05; ANOVA with Duncan’s multiple range test): lowercase for ethanol controls; uppercase for extracts. Asterisks indicate differences between ethanol controls and corresponding extract treatments (*, *p* < 0.05; **, *p* < 0.01; in Student’s *t*-test). Error bars represent standard error (n = 3). Regression analysis between ethanol and corresponding ethanolic extract treatments for each phytopathogen: (i) *F. oxysporum* f.sp. *radicis-lycopersici* (ethanol vs. *L. dentata* ethanolic extract: R = 0.977, R^2^ =0.954, *p* < 0.05); (ii) *V. dahliae* (ethanol vs. *T. vulgaris* ethanolic extract: R = 0.892; R^2^ = 0.795; not significant).

Up to 3% *v*/*v* ethanol, no inhibitory effect on the growth of *F. oxysporum* f.sp. *radicis-lycopersici* occurred in the in vitro assessment using PDB in the presence of the respective amount of ethanol (1–3%). However, an ethanol concentration of 4% *v*/*v* induced an inhibitory effect on the growth of this phytopathogenic fungus (*p* < 0.05, in Duncan’s multiple range test). During the application of 1% *v*/*v L. dentata* ethanolic extract, no statistically significant differences were observed compared to the ethanolic control. At 2% and 3% *v*/*v L. dentata* ethanolic extract setups, the growth of *F. oxysporum* f.sp. *radicis-lycopersici* significantly differed from the respective ethanol controls, indicating the contribution of lavender bioactive compounds to the inhibition of this phytopathogen. Application of lavender ethanolic extract at 4% *v*/*v* or above strongly affected *F. oxysporum* f.sp. *radicis-lycopersici* growth (Figure 3a). Hydrodistilled *L. latifolia* essential oil diluted in DMSO resulted in a MIC of 10 μg/mL during cultivation of *F. oxysporum* in PDB [44]. Growth of *V. dahliae* in PDB media in the presence of 1% and 2% *v*/*v* ethanol was similar to the non-ethanol-containing culture. On the other hand, the growth of the fungus was significantly inhibited during application of 3% and 4% *v*/*v* ethanol. In the presence of 1% *v*/*v T. vulgaris* ethanolic extract, no inhibition was noted. In comparison to the non-ethanol culture and the respective PDB media containing 2% and 3% *v*/*v* ethanol, the phytopathogenic fungus *V. dahliae* was gradually suppressed under the application of 2% and 3% *v*/*v T. vulgaris* ethanolic extracts. *T. vulgaris* ethanolic extracts of 3% and 4% *v*/*v* significantly affected fungal growth. However, inhibition in *V. dahliae* growth was more pronounced in 3% and 4% *v*/*v T. vulgaris* ethanolic extracts compared to the respective ethanol concentrations, indicating the suppressive effect of thyme against *V. dahliae* (Figure 3b). Interestingly, hydrodistilled *T. vulgaris* essential oil showed antifungal activity against *Verticillium fungicola*, reporting a MIC of 0.5–1.5 μL/mL and a MFC of 1.0–1.5 μL/mL [45].

### 3.3. Germination Index (GI) Tests

**Figure 4:**
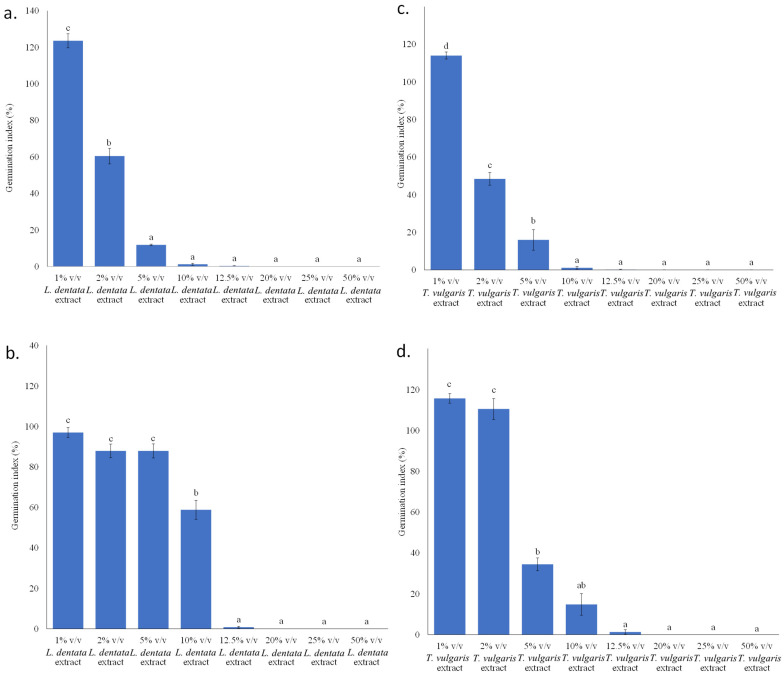
Germination index (GI) of *Solanum lycopersicum* cv. “Ace 55 VF” (**a**,**c**) and *Lepidium sativum* var. *sativum* (**b**,**d**) seeds at different concentrations of *L. dentata* (**a**,**b**) and *T. vulgaris* (**c**,**d**) extracts. A germination index of 100% corresponds to the control treatment (distilled water). Bars sharing no common letter indicate significant differences (*p* < 0.05; ANOVA with Duncan’s multiple range test). Error bars represent standard error (n = 3). Regression analysis: (i) for *S. lycopersicum* cv. “Ace 55 VF” (*L. dentata* vs. *T. vulgaris* ethanolic extract: R = 0.997; R^2^ = 0.993; *p* < 0.001); (ii) *L. sativum* var. *sativum* (*L. dentata* vs. *T. vulgaris*: R = 0.800; R^2^ = 0.640, not significant—ns); (iii) *L. dentata* (*S. lycopersicum* vs. *L. sativum*: R = 0.670, R^2^ = 0.449, ns); (iv) *T. vulgaris* (*S. lycopersicum* vs. *L. sativum*: R = 0.897, R^2^ = 0.805, ns).

A slight increase in the germination of tomato seeds was observed under the application of 1% *v*/*v L. dentata* extract (Figure 4a). A mοderate decrease occurred at 2% *v*/*v L. dentata* extract (*p* < 0.05, in Duncan’s multiple comparison test), while a low germination index (GI) was determined at 5% *v*/*v L. dentata* extract. Negligible or no germination was observed at 10% *v*/*v L. dentata* extract and thereafter. Less pronounced effects were observed in the *Lepidium sativum* germination tests (Figure 4b). No statistically significant differences were observed for 1, 2 and 5% *v*/*v L. dentata* extract. A decrease in GI occurred at 10% *v*/*v L. dentata* extract, while a further increase in the proportion of *L. dentata* extract resulted in severe inhibition of cress seeds. Ibáñez and Blázquez [46] reported that in vitro application of *L. angustifolia* essential oil up to 0.125 μL/mL did not exhibit a phytotoxic effect on tomato seed germination, while the seed germination index was reduced in a dose-dependent manner from 58 to 22% (by successively increasing dosage from 0.125 to 0.25, 0.5 and 1 μL/mL). Tomato seedling growth was not affected at the lowest dose application of *L. angustifolia* essential oil, whereas higher concentrations exerted a strong dose-dependent inhibitory effect. In contrast, application of *L. angustifolia* essential oil on cucumber seeds had no effect on seed germination, recording germination rates above 94% and only a slight reduction in radicle length was observed at higher doses during cucumber seedling growth. Vapors of *L. angustifolia* essential oil at a dose of 10 μL caused significant inhibition of germination and seedling growth of both barley and wheat, whereas concentrations of 30 and 90 μL completely suppressed germination and seedling development, respectively [47]. According to Terzić et al. [48], the application of 0.02% *L. angustifolia* essential oil increased the seed germination rate of three-year-old *Althea officinalis* seeds by 13% and enhanced seedling growth by 24–35%, while significantly suppressing seed pathogens, including a *Fusarium* sp.

A gradual decrease in tomato GI was observed by increasing *T. vulgaris* extract concentration from 1 to 5% *v*/*v*. Little to no germination was noted at 10% *v*/*v T. vulgaris* extract or above (Figure 4c). Regarding *Lepidium sativum* germination index, no inhibitory effect was observed up to 2% *v*/*v T. vulgaris* extract. However, germination index gradually declined from 2% to 12.5% *v*/*v T. vulgaris* extract setups, whereas no growth was observed above 12.5% *v*/*v T. vulgaris* extract (Figure 4d). Zheljazkov et al. [47] reported that vapors of *T. vulgaris* essential oil reduced germination and seedling growth of barley and wheat at 10 μL, whereas total inhibition was observed at concentrations of 30 and 90 μL. Moreover, the germination index of chickpea seeds was reported to not be affected by *Thymus saturejoides* essential oil at concentrations of 0.01–0.1%, whereas complete inhibition of germination was observed at 0.25% [49]. Thyme leaf extract at concentrations of 10–20% enhanced germination and growth of *Adenium arabicum* and *Euphorbia viguieri* seeds cultivated in peat and perlite under greenhouse conditions, while 30% thyme extract resulted in strong inhibition [50].

### 3.4. In Planta Assessment of Phytoprotective Activity of Lavandula dentata and Thymus vulgaris Extracts Against Fusarium oxysporum f.sp. radicis-lycopersici and Verticillium dahliae

**Figure 5:**
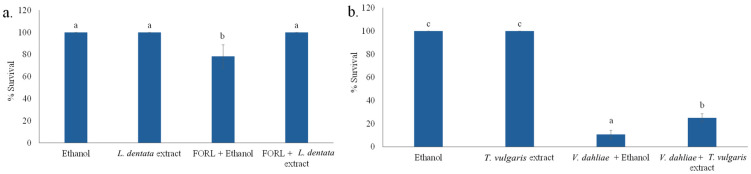
Survival (%) of tomato plants infected with *Fusarium oxysporum* f.sp. *radicis-lycopersici* (**a**) and *Verticillium dahliae* (**b**) following treatment with *Lavandula dentata* or *Thymus vulgaris* ethanolic extracts, compared to ethanol-treated controls. No infection was observed in non-inoculated plants (healthy control), whereas complete plant severity was recorded for pathogen-inoculated plants without treatment (pathogen-only control). Bars sharing no common letter indicate significant differences (*p* < 0.05; ANOVA with Duncan’s multiple range test). Error bars represent standard error (n = 4).

As expected, plants weekly treated with ethanol, lavender or thyme extract in the corresponding amount and used as controls remained unaffected. Moreover, in planta experiments demonstrated statistically significant phytoprotective effects of *L. dentata* extract against *F. oxysporum* f.sp. *radicis-lycopersici*. The survival rates of plants inoculated with *F. oxysporum* f.sp. *radicis-lycopersici* and treated with lavender extract were not affected during prolonged incubation time, whereas, at the same period, control plants showed significantly lower survival rates (Figure 5a). In addition, *T. vulgaris* extract appeared to delay tomato plants infection in a statistically significant manner, since, despite their high disease incidence values, greater statistically significant plant survival rates were recorded for pots inoculated with *V. dahliae* and treated with thyme extract, compared to the respective controls (Figure 5b). In planta experiments revealed the significant growth enhancement of sorghum plants treated with lavender essential oil compared to the control. At the molecular level, higher expression of three WRKY transcription factors (1, 19, and 45), jasmonate and ethylene-response factor 3 (JERF3) and eight defense-related genes were observed for pathogen-lavender essential oil treatment versus pathogen control as well as lavender essential oil treatment versus non-inoculated control [40]. Moreover, a commercial essential oil from thyme (1:9 *v*/*v*) inhibited *V. dahliae* growth during pot cultivation of twelve-month-old olive plants grown on naturally infested soil under semi-controlled conditions. A complete inhibition of *V. dahliae* was observed during the first two months as reported by Mulero-Aparicio et al. [51]. Similarly, application of 2% *Thymus* essential oil resulted in a slightly lower disease incidence regarding Verticillium wilt in olive plants compared to the control [27]. Lόpez-Escudero et al. [52] investigated the effect of organic amendments derived from *Thymus mastichina*, i.e., stems, leaves and flowers of this botanical species, on *V. dahliae* isolates V4 and V117 in cotton plants, reporting no viability of *V. dahliae* microsclerotia in soil and low disease incidence of cotton plants. Safari Motlagh and Hamdami [53] also reported that *T. vulgaris* ethanolic extract significantly reduced disease incidence (by 50% compared to the control) of rice plants grown under greenhouse conditions and inoculated with *Pyricularia oryzae*.

### 3.5. Lavandula dentata and Thymus vulgaris Extract Analysis

Bioactive substrates commonly identified in these aromatic plant species were also detected in the current study. In particular, non-volatile compounds, such as caffeoylquinic acid (mono-CQA), rosmarinic acid, an isorhamnetin-like flavonoid, a luteolin-O-glucuronide, myricetin-O-pentoside, and tricin [16,54,55], as well as eupatorin, quercetin, a luteolin/apigenin-type C-glycoside, and pentyl ferulate [56,57,58,59] were identified in the ethanolic extracts of lavender and thyme, respectively (Table 1 and Table 2). The differences in the observed antifungal activity of *L. dentata* and *T. vulgaris* extracts can be attributed to variations in their chemical composition. Although both extracts are rich in phenolics and flavonoids, thyme is particularly abundant in bioactive flavonoids, whereas lavender contains a diverse range of phenolic compounds, like rosmarinic acid. These secondary metabolites can contribute to antifungal activity by affecting membrane integrity, inhibiting spore germination and inducing oxidative stress in fungal cells [8,13]. Indeed, rosmarinic acid is among the most dominant phenolics in lavender [60,61], whereas luteolin-derived glycosides are considered among the most abundant polyphenols, commonly being the dominant flavonoid class in thyme extracts [62]. Interestingly, Steinkellner and Mammerler [63] reported that a low concentration of flavonoid compounds, including luteolin and myricetin, reduced the growth of *Fusarium oxysporum* f.sp. *lycopersici*, while microconidial germination was slightly stimulated. Martínez et al. [64] reported that chlorogenic acid, a phenolic secondary metabolite, reduced the mycelial growth of the phytopathogens *Fusarium solani*, *Sclerotinia sclerotiorum*, *Verticillium dahliae*, *Botrytis cinerea* and *Cercospora sojina* and completely suppressed their spore germination. Chlorogenic and caffeic acids also inhibited the growth of *F. graminearum* and *F. culmorum* and reduced mycotoxin production [65]. Furthermore, flavonoids, including apigenin and luteolin, inhibited the mycelial growth of *Verticillium albo-atrum* [66].

## 4. Conclusions

In this work, key plants of the Lamiaceae family abundant in the Mediterranean region were comparatively evaluated regarding their in vitro and in planta fungicidal activities against two major phytopathogenic fungi, i.e., *F. oxysporum* f.sp. *radicis-lycopersici* and *V. dahliae*. Application of *L. dentata* and *T. vulgaris* ethanolic extracts against *F. oxysporum* f.sp. *radicis-lycopersici* and *V. dahliae* respectively resulted in inhibitory effects at concentrations equal to or above 2% *v*/*v*. On the other hand, the application of such botanical extract concentrations did not affect the germination of both *Solanum lycopersicum* and *Lepidium sativum* seeds. In addition, common plant phenolic compounds were identified in both lavender and thyme extracts, which have previously been reported in the literature to exert an efficient phytoprotective action. These results provide new insights into the potential use of aromatic plants in sustainable phytoprotection as botanical fungicides, especially in the case of lavender that can serve as an efficient biocontrol agent, due to its enhanced antifungal properties and low phytotoxicity.

## Figures and Tables

**Table 1:** Putative identified compounds in *Lavandula dentata* ethanolic extract.

Putative Identified Compound	Precursor Mass (*m*/*z*)	Key MS/MS Fragments (*m*/*z*)	Proposed Formula	MSI Level *	Role/Origin
Caffeoylquinic acid (mono-CQA)	355.9	precursor: *m*/*z* 356.04 *m*/*z* 338.04: dehydration from the quinic-acid part *m*/*z* 253.00: a secondary fragment from a deeper cleavage/rearrangement	C_16_H_19_O_9_	3	Mono-CQA—a native lavender phenolic from plant’s phenylpropanoid pathway (caffeic acid part) plus shikimate-derived quinic acid [16,61,67]
Rosmarinic acid	428.6	precursor: *m*/*z* 428.60 ([M + H]^+^) *m*/*z* 392.88: loss of 2 H_2_O molecules → stepwise dehydration from a polyhydroxylated phenolic/hydroxyacid part *m*/*z* 374.95: loss of 3 H_2_O molecules → further dehydration of oxygen-rich phenolic part *m*/*z* 282.85: major bond cleavage consistent with ester/ether-type dissociation yielding a caffeic-acid-related/conjugated aromatic fragment *m*/*z* 262.85: further neutral loss from the aromatic fragment *m*/*z* 208.51: smaller aromatic/hydroxyacid fragment from deeper cleavage, which is indicative of multiple oxygenated aromatic subunits *m*/*z* 148.68: low-mass phenolic ring fragment (“aromatic core” product ion) *m*/*z* 122.62: small aromatic cation fragment from breakdown of the phenolic part	C_18_H_16_O_8_	2	Rosmarinic acid—a plant secondary metabolite formed by the conjugation of two phenylpropanoid-derived units [54,55,68]
Luteolin-O-glucuronide	458.8	precursor: *m*/*z* 458.05 flavonoid conjugate *m*/*z* 439.9: H_2_O loss, from polyhydroxylated flavonoid *m*/*z* 290.5: flavonoid aglycone-type ion, characteristic of luteolin-related structure *m*/*z* 206.6: retro-Diels-Alder (RDA) fragment—a flavone backbone diagnostic ion *m*/*z* 122.6: small aromatic ring fragment—a common end-stage flavonoid breakdown product	C_21_H_18_O_12_	2	A well-reported Lavandula-derived flavonoid conjugate formed by glucuronidation of luteolin [54,67]
Myricetin-O-pentoside	453.4	precursor: *m*/*z* 453.35 ([M + H]^+^) *m*/*z* 318.47: loss of pentose-like sugar moiety/sugar-derived cleavage, resulting in the aglycone ion, typical for myricetin-type flavonol *m*/*z* 363.47: partial sugar loss for O-glycosides *m*/*z* 408.42: loss of a small oxygenated neutral CO_2_/CHO_2_ from the precursor or adducted precursor *m*/*z* 314.41: ion aglycone-related rearrangement/H-transfer product, detecting in polyhydroxy flavonols *m*/*z* 359.43; additional sugar plus small neutral combined loss/rearranged glycoside fragment *m*/*z* 274.54: secondary aglycone fragmentation flavonol ring scission/sequential CO, CO_2_, H_2_O type losses *m*/*z* 246.58: further aglycone breakdown polyphenolic A/B-ring cleavage products *m*/*z* 215.53: aromatic/phenolic fragment from aglycone fragmentation *m*/*z* 173.45/172.60/171.63: RDA/ring fragments from a highly hydroxylated flavonol backbone	C_20_H_18_O_12_	3	A myricetin-type flavonol O-glycoside—a lavender polyphenol derived from the phenylpropanoid–flavonoid pathway involved in antioxidant defense and UV protection [67,69]
Tricin	329.9	precursor: *m*/*z* 329.9 ([M + H]^+^) *m*/*z* 312.0: H_2_O loss or flavonoid OH *m*/*z* 206.7: characteristic flavone RDA fragment *m*/*z* 148.8: aromatic ring fragment, typical of a flavone aglycone pattern	C_17_H_14_O_7_	2	Methoxylated flavone produced via the phenylpropanoid pathway involved in antioxidant defense and UV protection [54,70]
Isorhamnetin-like flavonoid (O-methylated quercetin-type aglycone)	339.9	precursor: *m*/*z* 339.93: ([M + Na])^+^ *m*/*z* 321.94: dehydration from a phenolic/polyhydroxylated aromatic part *m*/*z* 226.92: conjugated aromatic fragment from the flavonoid core *m*/*z* 208.96: H_2_O loss from oxygenated aromatic fragment	C_16_H_12_O_7_	3	Flavonol—a polyphenolic plant secondary metabolite [54,71]

* MSI level: Metabolomics Standards Initiative level.

**Table 2:** Putative identified compounds in *Thymus vulgaris* ethanolic extract.

Putative Identified Compound	Precursor Mass (*m*/*z*)	Key MS/MS Fragments (*m*/*z*)	Proposed Formula	MSI Level *	Role/Origin
Eupatorin	344.8	precursor: *m*/*z* 344.75 [M + H]^+^ *m*/*z* 329.75: CH_3_ loss (demethylation)—it suggests at least one methoxy (–OCH_3_) substituent that is common in methoxylated flavonoids/phenolics *m*/*z* 311.73: loss of CH_3_ + H_2_O *m*/*z* 283.83: loss of acetic acid, which is seen from acetylated groups or rearrangements in oxygenated aromatics *m*/*z* 268.80: an additional CH_3_ loss—it is indicative of multiple methoxy sites or repeated demethylation channels	C_18_H_16_O_7_	2	Eupatorin is a methoxylated flavone of Lamiaceae plants, including thyme, with function as a defensive secondary metabolite with antioxidant and antimicrobial activity, contributing to plant protection against pathogens and environmental stress [72,73]
Luteolin/apigenin-type C-glycoside (orientin/isoorientin-like)	453.4	precursor: *m*/*z* 453.28: [M + NH_4_]^+^ *m*/*z* 436.29: loss of NH_3_—formation of the corresponding [M + H]^+^ ion *m*/*z* 435.06: minor ion indicative of a dehydration-type channel *m*/*z* 363.47: a signature neutral loss characteristic of C-glycoside-type cross-ring cleavage *m*/*z* 318.46: deeper cleavage consistent with a C-glycoside fragmentation channel *m*/*z* 274.53: secondary breakdown product from successive fragmentation of the aglycone/substituent system *m*/*z* 215.40/172.65/128.72: secondary ions from further fragmentation	C_21_H_22_O_10_	3	Flavonoid C-glycosides (orientin/isoorientin-type compounds) are biosynthesized via the phenylpropanoid–flavonoid pathway and they can act as protective antioxidants and contribute to defense against pathogens and environmental stress [53,74]
Pentyl ferulate	282.0	precursor: *m*/*z* 282.00: [M + NH4]^+^ *m*/*z* 265.01: loss of NH_3_ *m*/*z* 247.03: loss of NH_3_ plus H_2_O from the precursor or dehydration from the 265 ion *m*/*z* 176.99: fragment indicative of a phenylpropanoid/ferulate-type core ion *m*/*z* 149.01: aromatic fragment characteristic of methoxy/hydroxy substituted phenyl systems *m*/*z* 134.92: aromatic fragment arisen from further cleavage/dealkylation of the phenyl fragment series	C_15_H_20_O_4_	3	Pentyl ferulate is a phenylpropanoid ester, formed from ferulic acid and short-chain alcohols, with antioxidant and protective function, supporting plants defend against oxidative stress and pathogens [56,75]
Quercetin	302.6	precursor: *m*/*z* 302.73: [M + H]^+^ *m*/*z* 270.69: neutral loss commonly reported for flavonoids *m*/*z* 252.70: further fragmentation of the flavonoid core *m*/*z* 242.76: loss observed in oxygen-rich aromatics/flavonoids *m*/*z* 234.62: further ring fragmentation/rearrangement from the aglycone. *m*/*z* 224.60: additional cleavage within the flavonoid backbone *m*/*z* 182.75: major diagnostic cleavage indicative of flavonoid RDA/ring-cleavage-type *m*/*z* 154.82: aromatic fragment indicative of further breakdown of the polyhydroxylated flavonoid ring *m*/*z* 155.38: aromatic ion in the same diagnostic region regarding flavonol/polyphenol scaffold (RDA-related) *m*/*z* 148.58: further breakdown of aromatic core fragment—polyphenolic ring fragmentation *m*/*z* 144.72: aromatic fragment formed by ring cleavage/rearrangement in oxygenated aromatics	C_15_H_10_O_7_	2	Quercetin is a flavonol biosynthesized via the phenylpropanoid pathway and commonly found in thyme and other Lamiaceae spp., providing antioxidant activity and protect against UV stress, pathogens, and herbivores [56,74]
Putative oxygenated diterpenoid (abietane/terpenoid ester)	444.9	precursor: *m*/*z* 444.77 [M + H]^+^ *m*/*z* 428.68: O-loss *m*/*z* 412.42: multiple dehydration/oxidation-type losses from an oxygen-rich terpenoid skeleton *m*/*z* 398.43: CO/H_2_O-type losses in oxygenated terpenoids *m*/*z* 360.86: side-chain cleavage from a higher-mass terpenoid/ester framework *m*/*z* 350.46: a side-chain/backbone cleavage product *m*/*z* 340.68: cleavage of an ester/side-chain substituent *m*/*z* 206.62/190.70/146.89/122.63: secondary fragments from further breakdown	C_24_H_28_O_8_	3	A carnosic acid–family derivative belonging to abietane diterpenes biosynthesized by Lamiaceae spp. (including thyme) from the terpenoid pathway and acting as defensive antioxidants and antimicrobials to protect from oxidative stress, UV exposure, and pathogens [76]

* MSI level: Metabolomics Standards Initiative level.

## Data Availability

The original contributions presented in this study are included in the article. Further inquiries can be directed to the corresponding author.
